# Mechanism of Synergy Between Tetracycline and Quercetin Against Antibiotic Resistant *Escherichia coli*

**DOI:** 10.3389/fmicb.2019.02536

**Published:** 2019-11-22

**Authors:** Shaoqi Qu, Cunchun Dai, Zhangqi Shen, Qihe Tang, Haixia Wang, Bing Zhai, Li Zhao, Zhihui Hao

**Affiliations:** ^1^College of Veterinary Medicine, China Agricultural University, Beijing, China; ^2^Agricultural Bio-Pharmaceutical Laboratory, Qingdao Agricultural University, Qingdao, China

**Keywords:** *Escherichia coli*, antibiotic, quercetin, synergism, resistance, mechanism

## Abstract

Treatment of multi-drug resistant (MDR) *Escherichia coli* intestinal infections are being hampered by the presence of the *mcr*-1 (colistin) and *tet* (tetracycline) resistance genes in these strains. We screened seven traditional Chinese medicines for their ability to synergize with tetracycline to provide an effective new drug for the treatment of animal intestinal diseases caused by MDR *E. coli*. Our primary screen identified quercetin as a compound that reduced the minimum inhibitory concentration (MIC) of tetracycline against the *E. coli* standard test strain American Type Culture Collection (ATCC) 25922 and clinical isolates fourfold from 4 and 256 μg/mL to 1 and 64 μg/mL, respectively. Low levels of quercetin in combination with tetracycline were bactericidal for clinical *E. coli* isolates and after 24 h, the differences between this combination and each drug singly were 10^8^ CFU/mL. We used this combination therapy in a mouse infection model and found 100% survival after 48 h compared with <50% for each drug alone. This drug combination also synergized to disrupt the bacterial cell envelope resulting in increased permeability and cell lysis. These data demonstrate that combinatorial screening at low concentrations constitutes an efficient approach to identify clinically relevant quercetin/tetracycline combinations and is a valuable prototypical combination that has a high clinical potential against *E. coli* infections.

## Introduction

Antibiotics play an important role in reducing the morbidity and mortality related to bacterial illnesses in animals and humans. However, antibiotic abuse and overuse has led to the emergence of resistant bacteria and enabled the transmission of antibiotic resistance genes (Ewers et al., 2012; Campion and Morrissey, 2013). *E. coli* is an essential symbiont of the mammalian human gut providing a source of vitamin K and other essential compounds but it can be easily spread via food and water since it is a facultative anaerobe (Ewers et al., 2012; Wu et al., 2013). Importantly, antibiotic resistant strains of *E. coli* have emerged and can be incorporated into the intestinal microbiome. In contrast, development of novel antibiotics to keep pace with the changing resistome has been much slower and challenging (Kirst, 2013; Wright, 2017; Jeong et al., 2018). This has reduced the therapeutic options for drug-resistant bacterial infections (Ferri et al., 2017).

The flavones as well as other natural products can increase the effectiveness of some antibiotics with their co-administration (Livermore, 2011). Tetracycline is a common broad-spectrum antibiotic, which acts as an antibacterial by inhibiting protein synthesis by preventing the attachment of aminoacyl-tRNA to the ribosomal acceptor (A) site (Chopra and Roberts, 2001). This synergistic effect has been demonstrated for gallic acid/tetracycline and cinnamaldehyde/erythromycin combinations (Visvalingam et al., 2017). Quercetin is a pentahydroxyflavone and a natural polyphenolic flavonoid that possesses antibacterial, anti-oxidant, and vasoactive properties that include lowering blood pressure in clinical tests (Martini et al., 2004; Edwards et al., 2007; Hirai et al., 2010). Quercetin possesses inherent antibacterial properties against *E. coli* and can alter its membrane permeability leading to leakage of intracellular contents. More specifically, quercetin inhibits the NLRP3 inflammasome activation in epithelial cells triggered by *E. coli* O157: H7 (Xue et al., 2017). Other flavonoids have been shown to target DNA gyrase and influence bacterial membrane fluidity (Tsuchiya and Iinuma, 2000; Wu et al., 2013).

In the current study, we assessed the *in vitro* antimicrobial activities of seven active components of traditional Chinese medicine. We examined the effects of quercetin gallic acid, magnolol, chlorogenic acid, paeoniflorin, matrine, and fumarate in combination with tetracycline, oxytetracycline, chlortetracycline, doxycycline, ofloxacin, norfloxacin, ciprofloxacin, florfenicol, cefquinome, and ceftiofur against eight *E. coli* stains using minimum inhibitory concentrations (MICs), checkerboard tests, and time-kill assays. By employing this strategy, we discovered that quercetin combined with tetracycline was effective against multi-drug resistant (MDR) *E. coli*. We further studied the mechanism by which tetracycline and quercetin act synergistically and demonstrated that the tetracycline/quercetin combination is effective against *E. coli in vivo*.

## Materials and Methods

### Bacterial Strains and Chemicals

The *E. coli* strain ATCC 25922 used for quality control was obtained from the American Type Culture Collection (Manassas, VA, United States). Clinical test strains were swine manure isolates obtained from the China Agricultural University, Beijing (GZP8-8, GZP10-8, 12a4, 12e5, GZP13-4) and from human blood (II-119 and II-CX53) as previously described and served as representative MDR *E. coli* strains (Wang et al., 2017; Shen et al., 2018). These were cultured in Mueller–Hinton broth (MHB) and maintained on MH agar (Haibo Biological Technology, Qingdao, China).

Tetracycline, oxytetracycline, chlortetracycline, doxycycline, ofloxacin, norfloxacin, ciprofloxacin, florfenicol, cefquinome, ceftiofur and gallic acid, quercetin, magnolol, chlorogenic acid, paeoniflorin, matrine, and fumarate were purchased from the China Institute of Veterinary Drugs Control (Beijing, China). Tetracycline, ciprofloxacin, cefquinome, chlorogenic acid, and matrine were dissolved in sterile water. Ofloxacin, norfloxacin, florfenicol, ceftiofur, and chlorogenic acid were dissolved in 10% glacial acetic acid (Tianjin Fuyu Fine Chemicals, Tianjin, China). Quercetin and gallic acid were dissolved in dimethyl sulfoxide (DMSO) purchased from the Laiyang Kant Chemical (Yantai, China). Magnolol, paeoniflorin, and fumarate were dissolved in ethanol (Tianjin Fuyu, Tianjin, China). All antibiotic solutions were sterilized using 0.22 μm filters prior to use (Jiangsu Green Union Science Instrument, Jiangsu, China).

### Minimal Inhibitory Concentration Determination

Minimum inhibitory concentrations (MICs) were determined using broth microdilution as previously described (see below). In brief, 100 μL MHB/well was added to 96-well microplates and a series of dilutions were made using 50 μL aliquots of antibiotic and test compound. Each well then received an initial inoculum of 1 × 10^6^ CFU/mL. Each experiment included control wells containing DMSO and inoculum. The plates were incubated for 18 h at 37°C. Cell turbidity was measured using an automated plate reader (Shanghai Haorui Instrument, Shanghai, China). MIC scoring used Clinical Laboratory Standards Institute guidelines and reference values (CLSI, 2015).

### Antibiotic Synergism Tests

The checkerboard method was combined with a fractional inhibitory concentration (FIC) index to determine the interaction between antibiotics and quercetin. Antibiotics (C) that showed resistance to the *E. coli* test strains were chosen for a further synergistic interaction test with quercetin (D). Each antibiotic/quercetin association was checked in triplicate and repeated twice. Antibiotics and quercetin were twofold serially diluted in a 96-well plate and incubated as described above. The combined inhibitory effect was examined by calculating the FIC index for each combination: FIC of C = MIC of C in combination/MIC of C alone; FIC of D = MIC of D in combination/MIC of D alone; FIC index = FIC of C + FIC of D. The standards used for a judgment of synergism were FIC ≤ 0.5, synergism; 0.5 > FIC < 1, additive effect; 1 > FIC < 2, no effect; FIC > 2, antagonistic effect (Zhao et al., 2001; Langeveld et al., 2014).

### Time-Kill Assays

Tetracycline sensitive and resistant *E.coli* strains at 1 × 10^6^ CFU/mL were used to inoculate tubes containing 10 mL of MHB containing tetracycline and quercetin both at 0.5× MIC. Tubes containing only MHB were inoculated as controls. The bacterial suspensions were incubated at 37°C with moderate shaking for 0, 4, 8, 16, and 24 h and 1 mL of bacterial suspension was removed and serially diluted in MHB. Fifty microliters of each dilution was spotted on MH agar plates and the CFU were counted after incubating the plate overnight at 37°C (Andrea et al., 2004).

### Mouse Survival Model

Briefly, eight groups of specific pathogen-free (SPF) Kunming mice (*n* = 6 per group) weighing 18–22 g (Jinan Pengyue, Jinan, China) were infected intraperitoneally (i.p.) with 0.5 mL of bacterial strain 12a4 totaling 2 × 10^7^ CFU per mouse. One hour after infection, mice were also treated with a single i.p. dose of quercetin and tetracycline at 50 and 96 mg/kg, respectively. Controls for these experiments were animals given quercetin or tetracycline alone at the above dosages. The positive control group received the bacterial inoculum only. One group was treated with a dose of 96 mg/kg tetracycline, and survival was observed 24 h after infection and analyzed by non-parametric control. Colistin was administered at 7.5 mg/kg for another positive control group and both strains were sensitive. Mice were euthanized by cervical dislocation and organs were aseptically removed, homogenized, serially diluted, and plated on MacConkey agar and incubated at 37°C for 24 h for CFU counting. This study was approved by the Qingdao Agricultural University Animal Experiment Committee [license Number: SYXK (SD) 20180006] and the animals were maintained in accordance with Qingdao Agricultural University guidelines for the care and use of laboratory animals.

### Cell Membrane Tests

Bacterial cell membrane disruption was assessed by measuring alkaline phosphatase release using a commercial kit (Solaibao, Beijing, China) and β-galactosidase release determined using 2-nitrophenyl β-D-galactopyranoside hydrolysis and a colorimetric assay at 405 nm (Liu et al., 2017). ATP levels were determined using a commercial kit (Solaibao, Beijing, China). Membrane permeability of *E. coli* induced by quercetin and tetracycline was tested by propidium iodide (PI) uptake as previously described (Liu et al., 2017).

### Scanning Electron Microscopy

Bacterial cultures of *E. coli* ATCC 25922 at mid-log phase in Luria Bertani (LB) broth were collected and suspended in phosphate-buffered saline (PBS). Bacteria were then treated with sublethal concentrations of quercetin and tetracycline at 37°C for 1 h. The cells were fixed in 2.5% glutaraldehyde, washed with PBS, and dehydrated in a graded ethanol series for Scanning Electron Microscopy (SEM) analysis as previously described (Liu et al., 2017). SEM was carried out using an Scanning Electron Microscope (JEOL 7500F, Japan).

### Tetracycline Accumulation Assays

Accumulated tetracycline was measured as previously described (Oh and Jeon, 2015). Briefly, *E. coli* were grown overnight to late log phase in MH broth in the presence of quercetin at 0.5× MIC. Samples (1 mL) were centrifuged, washed with 100 mM Tris buffer pH 8, and suspended in 1 mL of the same buffer. The bacteria were then cultured in the presence of tetracycline (100 μg/mL) for 15 min and 1 mL 5 M HCl was added and the cells were boiled for 10 min. This procedure quantitatively converted the tetracycline to anhydrous tetracycline. The cooled sample was centrifuged to remove the cells debris and anhydrous tetracycline in the supernatant was measured at excitation/emission wavelengths of 400 and 520 nm, respectively. The amount of anhydrous tetracycline contained in these samples was determined using a standard curve from 0 to 100 mg tetracycline/L (Andrea et al., 2004; Oh and Jeon, 2015).

### ARG Detection

Quantification of expression of the *tet* (A), *tet* (B), *tet* (M), and *tet* (S) genes was performed using reverse transcription-PCR (RT-PCR). GAPDH serves as a standardized reference gene. PCR primers were synthesized by Shanghai Shenggong Biological Engineering Co., Ltd. (Shanghai, China). Total RNA from strains GZP08-8, 12a4, 12e5, and II-CX53 cultured in MHB with or without drug for 16–18 h was isolated using a commercial RNA extraction kit (Tiangen Biotech, Beijing, China) according to the user’s guide. RT-PCR was performed using a commercial kit (Solaibao, Beijing, China) with cycles of 30 s at 95°C and 40 cycles of 5 s at 95°C and 30 s at 60°C (Zhao et al., 2001).

### Statistical Analysis

Values were expressed as mean ± SD for each group. All analyses were performed using SPSS version 12.0 software package (Chicago, IL, United States). Differences were considered to be statistically significant at *p* < 0.05.

## Results

### Antimicrobial Susceptibility Testing

**FIGURE 1:**
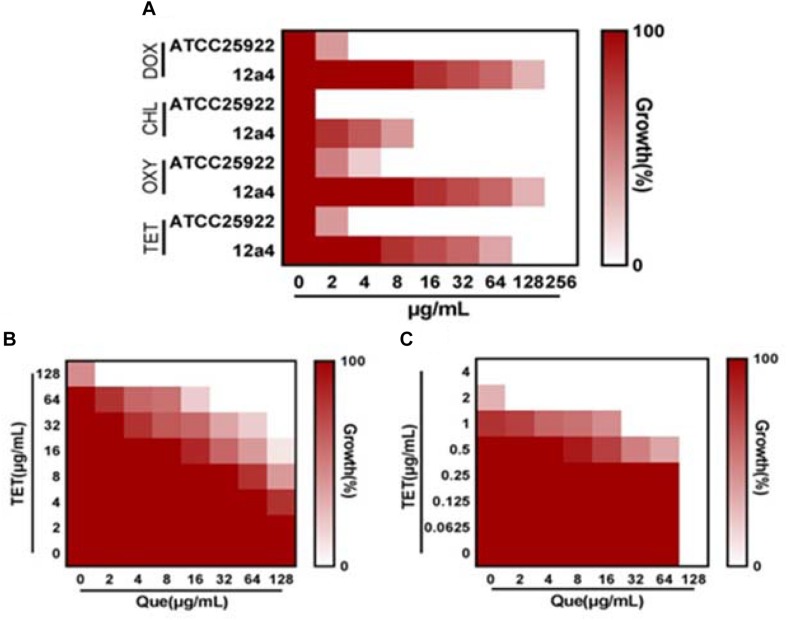
Quercetin potentiates the activity of tetracycline. **(A)** MIC determinations of the indicated antibiotics against tetracycline resistant (12a4) and sensitive (ATCC 25922) *E. coli* strains. Microdilution checkerboard analysis demonstrating the combined effect of quercetin and tetracycline against *E. coli* isolates **(B)** 12a4 MIC = 256 μg/mL and **(C)** ATCC 25922 MIC = 4 μg/mL. Heat plots are the average of three technical replicates.

We explored the antibacterial activities of 10 antibiotics against eight strains of *E. coli* using quercetin alone and in combination. The clinical isolates were all MDR strains but still most were sensitive to ceftiofur (MIC, 0.5 μg/mL) and least sensitive to cefquinome and norfloxacin (MIC, 1024 μg/mL) (Table 1). The MIC values of the seven test compounds derived from traditional Chinese medicine ranged from 128 to 2048 μg/mL indicating an overall weak effect (Table 2). However, there was a significant MIC decrease for tetracycline, oxytetracycline, chlortetracycline, and doxycycline with the addition of quercetin at 1–256 μg/mL. The FIC values ranged from 0.094 to 0.5 indicating strong synergism for the four tetracycline derivatives (Figure 1 and Table 3). Hence, the quercetin/tetracycline combination was selected for further investigation. We also selected the clinical MDR strain 12a4 for further testing because it displayed the typical tetracycline resistance profile of all the clinical strains and was representative of the synergy with quercetin among these strains.

**TABLE 1:** MIC values of antibiotics against the *E. coli* strains used in this study.

***E. coli***	**MIC of antibiotics (μg/mL)^*a*^**							
	**ATCC25922**	**GZP8-8 (pig)**	**GZP10-8 (pig)**	**12a4 (pig)**	**12e5 (pig)**	**GZP13-4 (pig)**	**II-119 (human)**	**II-CX53 (human)**
Tetracycline	4(S)	256(R)	128(R)	256(R)	256(R)	256(R)	256(R)	128(R)
Oxytetracycline	2(S)	16(R)	16(R)	16(R)	16(R)	256(R)	16(R)	512(R)
Chlortetracycline	8(S)	256(R)	256(R)	256(R)	128(R)	512(R)	512(R)	512(R)
Doxycycline	4(S)	128(R)	128(R)	128(R)	128(R)	64(R)	128(R)	128(R)
Ofloxacin	2(S)	32(R)	16(I)	32(R)	16(I)	32(R)	128(R)	16(I)
Norfloxacin	4(S)	256(R)	128(R)	128(R)	128(R)	256(R)	128(R)	128(R)
Ciprofloxacin	4(S)	128(R)	32(R)	32(R)	16(I)	32(R)	128(R)	32(R)
Florfenicol	8(S)	32(R)	32(R)	64(R)	32(R)	16(I)	256(R)	256(R)
Cefquinome	8(S)	1024(R)	16(I)	32(R)	8(S)	1024(R)	512(R)	16(I)
Ceftiofur	1(S)	512(R)	1(S)	2(S)	0.5(S)	512(R)	128(R)	1(S)

*^*a*^S, sensitive; I, intermediary; R, resistant.*

**TABLE 2:** MIC values of drug against the *E. coli* strains used in this study.

***E. coli***	**MIC of drug (μg/mL)**						
	**Gallic acid**	**Quercetin**	**Magnolol**	**Chlorogenic acid**	**Paeoniflorin**	**Matrine**	**Fumarate**
ATCC25922	128	128	256	128	64	128	256
GZP8-8 (pig)	512	256	1024	1024	2048	1024	1024
GZP10-8 (pig)	512	512	1024	512	2048	1024	2048
12a4 (pig)	512	256	512	1024	512	2048	1024
12e5 (pig)	256	512	1024	1024	2048	2048	2048
GZP13-4 (pig)	256	128	1024	1024	2048	2048	2048
II-119 (human)	256	512	512	1024	512	512	2048
II-CX53 (human)	512	512	512	512	2048	1024	1024

**TABLE 3:** Enhancement of antibiotic effectiveness with quercetin expressed using the fractional inhibitory concentration (FIC) index.

	**FIC index^a^**							
**Antibiotic**	***E. coli* strain**							
	**ATCC25922**	**GZP8-8**	**GZP10-8**	**12a4**	**12e5**	**GZP13-4**	**II-119**	**II-CX53**
Tetracycline	0.5	0.375	0.094	0.375	0.312	0.5	0.125	0.5
Oxytetracycline	0.265	0.5	0.265	0.254	0.258	0.281	0.187	0.252
Chlortetracycline	0.254	0.28	0.375	0.5	0.375	0.254	0.375	0.5
Doxycycline	0.5	0.156	0.187	0.5	0.25	0.281	0.187	0.258
Ofloxacin	0.75	1	2	1	–^b^	1	1.5	1
Norfloxacin	1	1.5	0.75	1.5	1	1	1.5	2
Ciprofloxacin	1.5	1	1.5	2	–	1	2	1
Florfenicol	1	1	1	1.5	0.75	–	1.5	0.75
Cefquinome	0.75	1.5	–	–	–	1	1	1
Ceftiofur	0.75	0.75	–	–	2	0.75	1	2

*^*a*^FIC ≤ 0.5, synergism; 0.5 > FIC < 1, additive effect; 1 > FIC < 2, no effect; FIC > 2, antagonistic effect. ^*b*^Not tested.*

### Time-Kill Studies

**FIGURE 2:**
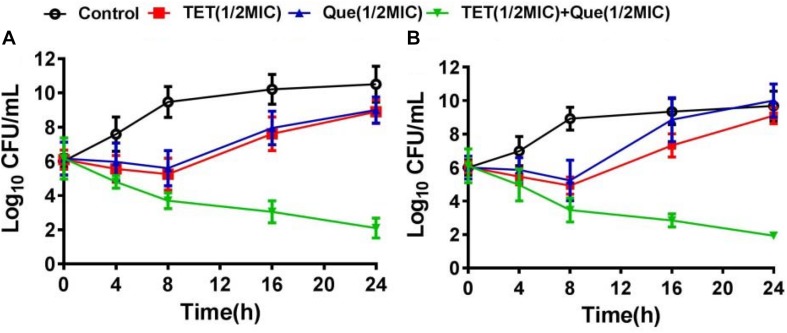
Time kill-curves for tetracycline in **(A)** resistant (12a4) and **(B)** sensitive (ATCC 25922) *E. coli* strains. Bacteria were grown in the presence of tetracycline (128 μg/mL for resistant strains and 2 μg/mL for susceptible strains) and in the presence and absence of quercetin at 128 μg/mL (0.5× MIC). 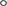, control; 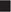, tetracycline (0.5× MIC); 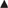, quercetin 128 μg/mL (0.5× MIC); 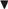, tetracycline (0.5× MIC) plus quercetin at 128 μg/mL. Error bars indicate standard deviations. The experiments were performed three times. Data are expressed as mean ± standard deviation.

We used strains 12a4 and ATCC 25922 (control) in a series of time kill studies with tetracycline and quercetin at 0.5× MIC. Bacteria exposed to each drug alone displayed almost identical kill curves. In contrast, in both the tetracycline sensitive and resistant isolates the tetracycline/quercetin combination, cell viability decreased more than eightfold by 24 h. In addition, by this time the cells exposed to single compounds had recovered and were comparable to the control cultures not exposed to drugs (Figure 2). Therefore, the bactericidal activity of tetracycline was significantly enhanced when in combination with quercetin.

### Quercetin Synergistically Enhances Tetracycline Activity *in vivo*

**FIGURE 3:**
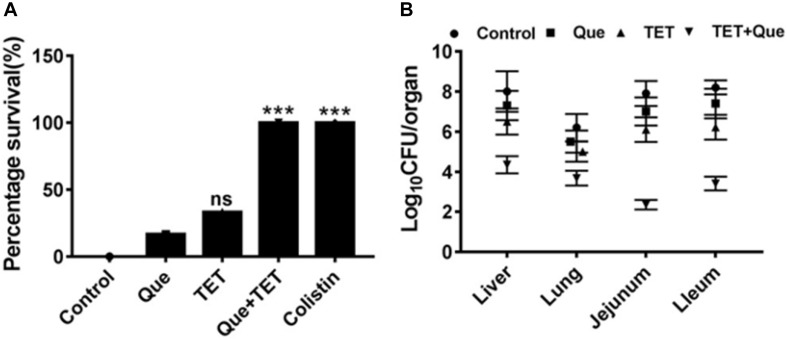
SPF mouse survival after i.p. injection of *E. coli* strain 12a4. **(A)** Survival of mice treated with quercetin (Que) 50 mg/kg alone and in combination with tetracycline (96 mg/kg) after 48 h. Colistin served as a positive control since both strains were sensitive. **(B)** CFU counts in the indicated organs taken from mice used for the experiments in **A**. ns, not significant; ^∗∗∗^*p* < 0.001 (determined by a two-sample *t*-test).

To demonstrate the efficacy of this combination therapy we adopted a mouse i.p. injection model of *E. coli* infection to our system using SPF Kunming mice. Mouse mortality after intraperitoneal injection of *E. coli* strain 12a4 was 90% after 48 h. Interestingly, single doses of either tetracycline or quercetin gave survival rates of <50%. In contrast, there was a 100% survival rate of mice receiving combination therapy of quercetin and tetracycline equivalent to the colistin treatment group (Figure 3A). When we examined the degree of bacterial proliferation in tissues of these mice, in all the organs we examined, the CFU values were significantly less than for the mice receiving monotherapies (Figure 3B). Together these data indicate that quercetin in combination with tetracycline displayed significant synergism *in vivo* resulting in a better therapeutic effect. This provides a practical foundation for clinical testing.

### Membrane Permeability

**FIGURE 4:**
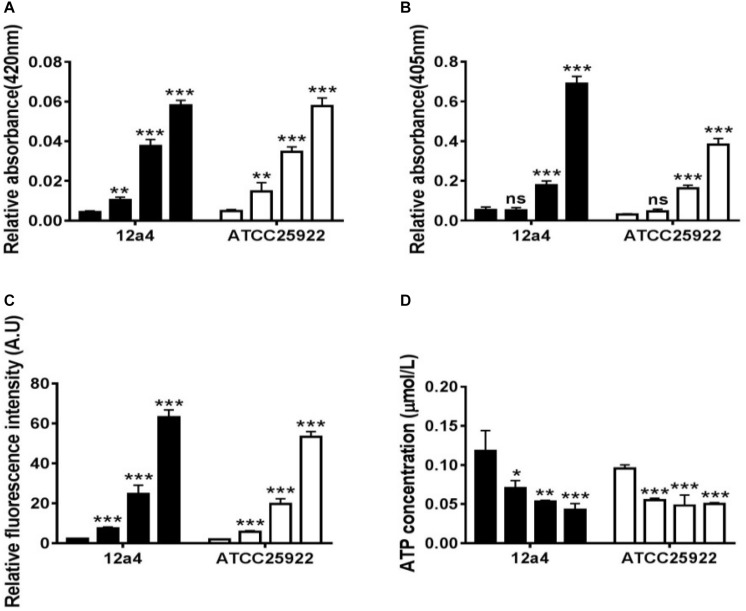
Quercetin and tetracycline combine to disrupt bacterial cell membrane integrity. From left to right, the bars represent control, quercetin, and tetracycline as individual compounds and quercetin/tetracycline in combination for the MDR strain 12a4 and the susceptible control strain ATCC 25922. The charts indicate the presence of the following in the culture medium after exposure to the indicated compounds: **(A)** β-galactosidase, **(B)** alkaline phosphatase, **(C)** ATP, and **(D)** propidium iodide. ns, not significant; ^∗^*p* < 0.05; ^∗∗^*p* < 0.01; ^∗∗∗^*p* < 0.001 (determined by a two-sample *t*-test). The results are shown as the mean and standard deviation of three independent experiments.

**FIGURE 5:**
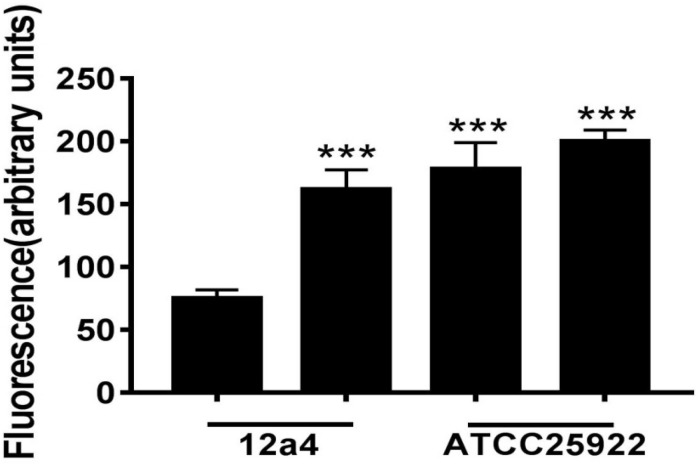
Tetracycline accumulation and release by *E. coli* in the presence and absence of quercetin. Bacterial suspensions were incubated in triplicate for 15 min with tetracycline (100 μg/mL) with or without quercetin pre-incubation at 128 μg/mL. Error bars indicate standard deviations. ^∗∗∗^*p* < 0.001 (determined by a two-sample *t*-test). The results are shown as the mean and standard deviation of three independent experiments.

**FIGURE 6:**
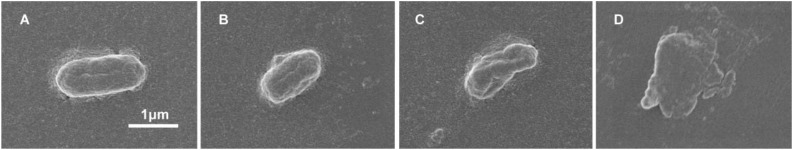
Disruption of cell envelope integrity by quercetin and tetracycline. SEM micrographs of *E. coli* strain 12a4 exposed to **(A)** media alone, **(B)** quercetin 0.5× MIC, **(C)** tetracycline 0.5× MIC, and **(D)** combination therapy at 0.5× MIC. Magnification = 15,000×.

We wanted to further investigate mechanisms that led to the synergistic action of quercetin. When we treated cells with quercetin, in the presence tetracycline the levels of β-galactosidase and alkaline phosphatase were significantly elevated (Figures 4A,B) indicative of cellular membrane disruption. This disruption was further illustrated using a PI uptake assay. The combination therapy also generated a significant increase in fluorescence intensity due to PI uptake (Figure 4C). ATP released outside the cell was also increased with the combination therapy but the results were less dramatic (Figure 4D). Tetracycline uptake was also increased in the presence of quercetin. The tetracycline resistance strain was converted to tet-sensitivity to a level comparable to the tet-sensitive control strain and was a further indicator of the disruption of membrane integrity (Figure 5). These bacteria that were exposed to the drug combination were also generally lysed and deformed (Figure 6). Together, these data indicate that the combination therapy produces profound ultrastructural changes leading to an increase in permeability and a weakening of the cell envelope.

**FIGURE 7:**
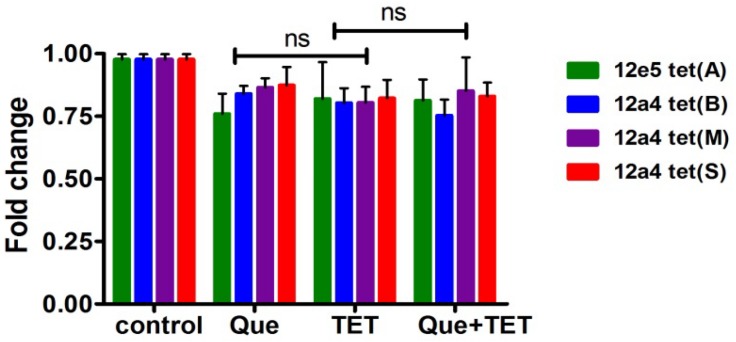
Steady state mRNA levels of selected *tet* genes in the presence of quercetin and tetracycline at 0.5× MIC levels of each drug were used as indicated. The fold change in mRNA levels was calculated based on the internal reference gene GAPDH, quantified by RT-PCR. The results are shown as the mean and standard deviation of three independent experiments. ns, not significant.

**FIGURE 8:**
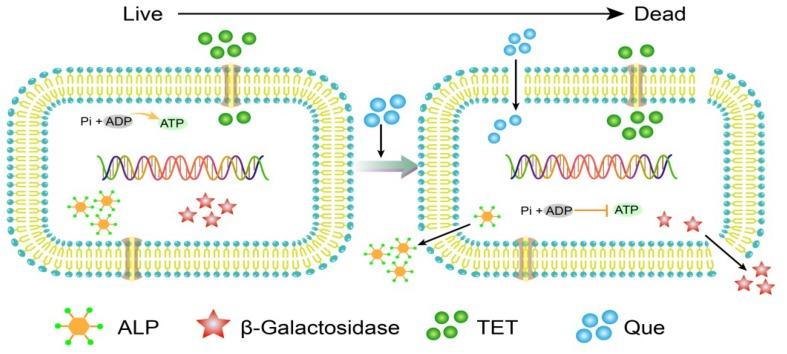
A model of quercetin and tetracycline combination therapy on *E. coli* cells.

### Gene Expression

The *tet* (A) and (B) genes encode efflux pumps that can protect cells by effective efflux action while tet (M) and (S) encode ribosomal protection proteins. We examined mRNA levels of these genes when cells were exposed to the regimen of quercetin, tetracycline, and the combination. The effect of quercetin on the expression of ribosomal protective and efflux pump genes was evaluated using strains 12a4 and 12e5, respectively. However, we found no significant differences in the steady state mRNA levels in any of the genes and this was independent of treatment (Figure 7).

## Discussion

Multi-drug resistant bacteria are an enormous challenge to the healthcare system and resistance can be found in both animal and human isolates (Chopra and Roberts, 2001). *E. coli* MDR isolates include pathogenic variants from food animals, meat, fish, seafood, and humans and carry transferable resistance genes (Tadesse et al., 2012). The abuse of antimicrobial agents in the treatment of bacterial infections has resulted in a significant reduction of clinical efficacy of previously effective antimicrobials. In our study, we investigated the effect of combining traditional Chinese medicines with antibiotics and found that quercetin lowered the MIC for tetracyclines for all the eight *E. coli* clinical isolates we tested. This combination also was significantly synergistic. In the presence of quercetin, the MIC of tetracycline was reduced 4–16-fold depending on the strain. The antibacterial effects of quercetin alone were consistent with experiments using *Actinobacillus actinomycetemcomitans* and *Fusobacterium nucleatum* and other oral bacterial pathogens. Moreover, the bactericidal effect of tetracycline was achieved at a very low concentration in combination with quercetin and is consistent with reports of using sub-inhibitory antibiotic concentrations with other natural products. For example, a twofold MIC for ciprofloxacin (0.25–0.12 μg/mL) was achieved against *Staphylococcus aureus* ATCC 29213 when tested in combination with piperine at 12.5 and 25 μg/mL (Khan et al., 2006). The efficacy of 5′-methoxyhydnocarpin strongly potentiated the action of berberine and other NorA substrates against *S. aureus* (Stermitz et al., 2000).

In addition to quercetin, the flavones are generally disruptors of bacterial cell walls. For instance, another flavone baicalein can synergize with tetracycline to interfere with cell wall integrity by binding directly to peptidoglycan (Zhao et al., 2001). In addition, baicalein restores the effectiveness of tetracycline toward methicillin-resistant *S. aureus* (MRSA) by inhibiting Tet K-mediated tetracycline efflux and inhibiting Tet M and other pumps. This suggests that quercetin is an efflux pump inhibitor (Michaela et al., 2014). We observed this indirectly in our experiments. The combination therapy resulted in collapsed and wrinkled cells indicating a gradual decrease in cell envelope integrity. We also observed an increase in fluorescence intensity due to PI uptake and DNA binding of tetracycline. In addition, we demonstrated loss of membrane integrity by demonstrating leaking of β-galactosidase, alkaline phosphatase, and ATP into the culture medium of treated cells as has been previously described (Nityakalyani et al., 2010) (Figure 8). *E. coli* and *S. aureus* treated with cinnamaldehyde exhibited numerous abnormalities including cytoplasmic membrane separation from the cell wall, cell membrane lysis, and cytoplasmic content leakage (Shen et al., 2015). Quercetin has been shown to inhibit nucleotide biosynthesis and ATP activity (Brvar et al., 2010) and we found a measurable decrease in intracellular ATP levels with combination therapy.

Flavonoids are less toxic to humans and animals because they are widely distributed in edible plants (Ofer et al., 2005). Quercetin is also used as a dietary supplement (250–500 mg three times a day) for therapeutic purposes. In cytotoxicity tests, proliferation of Chinese hamster ovary cells was inhibited by 50% after 24 h exposure to quercetin at 24 mg/L, for mouse fibroblasts at 36 mg/L, and normal rat kidney cells at 21 mg/L (Ngomuo and Jones, 1996). Quercetin exhibited a low IC_50_ value after 72 h of incubation with L929 cells (41.8 mg/L) (Kuhlmann, 1998). Determination of synergy or potentiation *in vitro* might not be reflected *in vivo* because of the potential failure to achieve synergistic levels of drugs in target tissues, differences in plasma protein binding, and metabolism of the drug. Importantly, we found that the combination therapy of tetracycline and quercetin at 0.5× MIC levels was effective in a mouse model of *E. coli* infection. This combination significantly enhanced mouse survival against bacterial challenge over each of the drugs given individually.

## Conclusion

We found that quercetin can greatly enhance the sensitivity of *E.coli* to tetracycline. Moreover, preliminary data show that quercetin and tetracycline act in synergy by altering cell membrane permeability of drug-resistant *E. coli*. The combination of quercetin and tetracycline did not result in significant cytotoxicity in mice thereby achieving the dual therapeutic requirements of a high antibacterial effect. Therefore, the combined use of tetracycline and quercetin should be further examined in clinical studies.

## Data Availability Statement

The datasets generated for this study are available on request to the corresponding author.

## Ethics Statement

The animal study was reviewed and approved by the Qingdao Agricultural University Animal Experiment Committee.

## Author Contributions

CD, ZS, and ZH designed the experiments. QT, HW, and BZ inspected the experiments. CD, SQ, and LZ executed most of the experiments. All authors analyzed the data and contributed to write the manuscript.

## Conflict of Interest

The authors declare that the research was conducted in the absence of any commercial or financial relationships that could be construed as a potential conflict of interest.

## References

[B1] Andrea S. R. Anna R. B. Francesco G. (2004). Epigallocatechin-gallate enhances the activity of tetracycline in staphylococci by inhibiting its efflux from bacterial cells. *Antimicrob. Agents Chemother.* 48 1968–1973. 10.1128/aac.48.6.1968-1973.2004 15155186 PMC415601

[B2] Brvar M. Perdih A. Oblak M. Masic L. P. Solmajer T. (2010). In silico discovery of 2-amino-4-(2,4-dihydroxyphenyl)thiazoles as novel inhibitors of DNA gyrase B. *Bioorg. Med. Chem. Lett.* 20 958–962. 10.1016/j.bmcl.2009.12.060 20045642

[B3] Campion E. W. Morrissey S. (2013). A different model–medical care in Cuba. *N. Engl. J. Med.* 368 297–299. 10.1056/nejmp1215226 23343058

[B4] Chopra I. Roberts M. (2001). Tetracycline antibiotics: mode of action, applications, molecular biology, and epidemiology of bacterial resistance. *Microbiol. Mol. Biol. Rev.* 65 232–260. 10.1128/mmbr.65.2.232-260.2001 11381101 PMC99026

[B5] CLSI (2015). *Methods for Dilution Antimicrobial Susceptibility Tests for Bacteria That Grow Aerobically.* Wayne, PA: CLSI.

[B6] Edwards R. L. Lyon T. Litwin S. E. (2007). Quercetin reduces blood pressure in hypertensive subjects. *J. Nutr.* 137 2405–2411. 10.1093/jn/137.11.2405 17951477

[B7] Ewers C. Bethe A. Semmler T. (2012). Extended-spectrum ß-lactamase-producing and AmpC-producing *Escherichia coli* from livestock and companion animals, and their putative impact on public health: a global perspective. *Clin. Microbiol. Infect.* 18 46–65. 10.1111/j.1469-0691.2012.03850.x 22519858

[B8] Ferri M. Ranucci E. Romagnoli P. Giaccone V. (2017). Antimicrobial resistance: a global emerging threat to public health systems. *Crit. Rev. Food Sci. Nutr.* 57 2857–2876. 10.1080/10408398.2015.1077192 26464037

[B9] Hirai I. Okuno M. Katsuma R. Arita N. Tachibana M. Yamamoto Y. (2010). Characterisation of anti-*Staphylococcus aureus* activity of quercetin. *Int. J. Food Sci. Technol.* 45 1250–1254. 10.1111/j.1365-2621.2010.02267.x

[B11] Jeong S. Kim J. O. Yoon E. J. Bae I. K. Lee W. Lee H. (2018). Extensively drug-resistant *Escherichia coli* sequence type 1642 carrying an IncX3 plasmid containing the blaKPC-2 gene associated with transposon Tn4401a. *Ann. Lab. Med.* 38 17–22. 10.3343/alm.2018.38.1.17 29071814 PMC5700142

[B13] Khan I. A. Mirza Z. M. Kumar A. Verma V. Qazi G. N. (2006). Piperine, a phytochemical potentiator of ciprofloxacin against *Staphylococcus aureus*. *Antimicrob. Agents Chemother.* 50 810–812. 10.1128/aac.50.2.810-812.2006 16436753 PMC1366922

[B14] Kirst H. A. (2013). Developing new antibacterials through natural product research. *Expert Opin. Drug Discov.* 8 479–493. 10.1517/17460441.2013.779666 23480029

[B15] Kuhlmann M. K. (1998). Reduction of cisplatin toxicity in cultured renal tubular cells by the bioflavonoid quercetin. *Arch. Toxicol.* 72 536–540. 10.1007/s002040050539 9765070

[B16] Langeveld W. T. Veldhuizen E. J. A. Burt S. A. (2014). Synergy between essential oil components and antibiotics: a review. *Crit. Rev. Microbiol.* 40 76–94. 10.3109/1040841X.2013.763219 23445470

[B17] Liu Y. Ding S. Dietrich R. Martlbauer E. Zhu K. (2017). A biosurfactant-inspired heptapeptide with improved specificity to kill MRSA. *Angew. Chem. Int. Ed. Engl.* 56 1486–1490. 10.1002/anie.201609277 28106348

[B19] Livermore D. M. (2011). Discovery research: the scientific challenge of finding new antibiotics. *J. Antimicrob. Chemother.* 66 1941–1944. 10.1093/jac/dkr262 21700626

[B20] Martini N. D. Katerere D. R. Eloff J. N. (2004). Biological activity of five antibacterial flavonoids from *Combretum erythrophyllum* (combretaceae). *J. Ethnopharmacol.* 93 207–212. 10.1016/j.jep.2004.02.030 15234754

[B21] Michaela W. Alina lulia C. Andreas O. Dagmar Z. Caroline M. Catherine S. (2014). Small cationic antimicrobial peptides delocalize peripheral membrane proteins. *Proc. Natl. Acad. Sci. U.S.A.* 111:5075. 10.1073/pnas.1319900111 24706874 PMC3986158

[B22] Ngomuo A. J. Jones R. S. (1996). Cytotoxicity studies of quercetin, shikimate, cyclohexanecarboxylate and ptaquiloside. *Vet. Hum. Toxicol.* 38 14–18. 8825742

[B23] Nityakalyani S. Peter J. Bernhard J. U. Martina W. Katja Z. Jessica S. (2010). Peptidomimetic antibiotics target outer-membrane biogenesis in *Pseudomonas aeruginosa*. *Science* 327 1010–1013. 10.1126/science.1182749 20167788

[B24] Ofer M. Wolffram S. Koggel A. Spahn-Langguth H. Langguth P. (2005). Modulation of drug transport by selected flavonoids: involvement of P-gp and OCT? *Eur. J. Pharm. Sci.* 25 263–271. 10.1016/j.ejps.2005.03.001 15911222

[B25] Oh E. Jeon B. (2015). Synergistic anti-campylobacter jejuni activity of fluoroquinolone and macrolide antibiotics with phenolic compounds. *Front. Microbiol.* 6:1129. 10.3389/fmicb.2015.01129 26528273 PMC4602130

[B26] Shen S. X. Zhang T. H. Yuan Y. Lin S. Y. Xu J. Y. Ye H. Q. (2015). Effects of cinnamaldehyde on *Escherichia coli* and *Staphylococcus aureus* membrane. *Food Control* 47 196–202. 10.1016/j.foodcont.2014.07.003

[B27] Shen Y. Wu Z. Wang Y. Zhang R. Zhou H. W. Wang S. (2018). Heterogeneous and flexible transmission of mcr-1 in hospital-associated *Escherichia coli*. *mBio* 3:e943-18. 10.1128/mBio.00943-18 29970465 PMC6030559

[B28] Stermitz F. R. Lorenz P. Tawara J. N. Zenewicz L. A. Lewis K. (2000). Synergy in a medicinal plant: antimicrobial action of berberine potentiated by 5′-methoxyhydnocarpin, a multidrug pump inhibitor. *Proc. Natl. Acad. Sci. U.S.A.* 97 1433–1437. 10.1073/pnas.030540597 10677479 PMC26451

[B30] Tadesse D. A. Zhao S. Tong E. Ayers S. Singh A. Bartholomew M. J. (2012). Antimicrobial drug resistance in *Escherichia coli* from humans and food animals. *U.S. Emerg. Infect. Dis.* 18 741–749. 10.3201/eid1805.111153 22515968 PMC3358085

[B31] Tsuchiya H. Iinuma M. (2000). Reduction of membrane fluidity by antibacterial sophoraflavanone G isolated from *Sophora exigua*. *Phytomedicine* 7 161–165. 10.1016/s0944-7113(00)80089-6 10839220

[B32] Visvalingam J. Palaniappan K. Holley R. A. (2017). In vitro enhancement of antibiotic susceptibility of drug resistant *Escherichia coli* by cinnamaldehyde. *Food Control* 79 288–291. 10.1016/j.foodcont.2017.04.011

[B33] Wang Y. Tian G. B. Zhang R. Shen Y. Tyrrell J. M. Huang X. (2017). Prevalence, risk factors, outcomes, and molecular epidemiology of mcr-1-positive *Enterobacteriaceae* in patients and healthy adults from China: an epidemiological and clinical study. *Lancet Infect. Dis.* 17 390–399. 10.1016/S1473-3099(16)30527-8 28139431

[B34] Wright G. D. (2017). Opportunities for natural products in 21(st) century antibiotic discovery. *Nat. Prod. Rep.* 34 694–701. 10.1039/c7np00019g 28569300

[B35] Wu T. Zang X. X. He M. Y. (2013). Structure–activity relationship of flavonoids on their anti-*Escherichia coli* activity and inhibition of DNA gyrase. *J. Agric. Food Chem.* 61 8185–8190. 10.1021/jf402222v 23926942

[B36] Xue Y. Du M. Zhu M. J. (2017). Quercetin suppresses NLRP3 inflammasome activation in epithelial cells triggered by *Escherichia coli* O157:H7. *Free Radic. Biol. Med.* 108 760–769. 10.1016/j.freeradbiomed.2017.05.003 28476502

[B37] Zhao W. H. Hu Z. Q. Okubo S. Hara Y. Shimamura T. (2001). Mechanism of synergy between epigallocatechin gallate and beta-lactams against methicillin-resistant *Staphylococcus aureus*. *Antimicrob. Agents Chemother.* 45 1737–1742. 10.1128/aac.45.6.1737-1742.2001 11353619 PMC90539

